# SARS-CoV-2 post-infective myocarditis: the tip of COVID-19 immune complications?

**DOI:** 10.1186/s13613-020-00717-0

**Published:** 2020-07-23

**Authors:** Pierre Tissières, Jean-Louis Teboul

**Affiliations:** 1 Pediatric Intensive Care, AP-HP Paris Saclay University, 78, Rue du Général Leclerc, 94275 Le Kremlin-Bicêtre, France; 2 Institute of Integrative Biology of the Cell, CNRS, CEA, Univ. Paris Saclay, Gif-sur-Yvette, France; 3 Medical Intensive Care, AP-HP Paris Saclay University, Bicêtre Hospital, Le Kremlin-Bicêtre, France

**Keywords:** COVID-19, SARS-CoV-2, MIS-C, Myocarditis, Kawasaki disease, Post-infective complications

## Abstract

Recent paediatric cases of acute myocarditis following a SARS-CoV-2 infection have raised the possibility of post-infective complications of COVID-19. This short editorial is reviewing current understanding of this new complication, its pathophysiology, diagnosis and therapeutic strategy.

## COVID-19 post-infective acute myocarditis, an unrecognized complication?

Recent descriptions of a short outbreak of acute myocarditis in otherwise healthy children raise the hypothesis of additional critical complications of SARS-CoV-2 infection [1–6]. Grimaud et al. just reported in *Annals of Intensive Care*, a series of 20 critically ill children with shock admitted, during a 12-day period, in four pediatric intensive care units in the Paris urban area [1]. This outbreak occurred after 4–6 weeks of strict lockdown. All children had acute myocardial dysfunction requiring cardiovascular support. This study confirmed similar observations from United Kingdom, France and Switzerland [2–6]. In the Grimaud et al. study, 19 out of 20 children had either positive SARS-CoV-2 detection by quantitative PCR or positive serology. The remaining child had a typical SARS-CoV-2 chest tomography scan. Note that all children had abdominal symptoms [1].

Acute heart failure is clearly a dissonant clinical feature of COVID-19 infection in children that is known to be less frequent and less severe than in adults and with very low mortality [7, 8]. Besides its unusual nature, the main characteristic of COVID-19 acute myocarditis is its association with major multisystem inflammatory syndrome, mimicking a well-known pediatric entity, the Kawasaki disease. Currently three case definitions issued from the World Health Organization, the Centers for Disease Control and Prevention and the Royal College of Paediatrics and Child Health related to this emerging inflammatory condition during COVID-19 pandemic exist. Those definitions identify the multisystem inflammatory syndrome in children (MIS-C) or the Pediatric Inflammatory Multisystem Syndrome (PIMS) [4]. All three case definitions include either partial or full criteria for Kawasaki disease and evidence of COVID-19 diagnosis (clinical and/or biological—serology/PCR). Kawasaki disease’s, which affect mostly young children of less than 5 years of age, has typical clinical features including: (1) prolonged fever, (2) conjunctivitis, (3) dry cracked lips, ((4) cervical adenopathy (5) diffuse skin rash involving the trunk and extremities, subsequent desquamation of the tips of the toes and fingers, and 6) edema. In addition to classical manifestations of Kawasaki disease, MIS-C patients, who are much older, display digestive symptoms, shock and myocardial involvement more frequently [9]. Kawasaki disease pathophysiology refers to a systemic arteritis with the most severe complication being coronary aneurysm. Kawasaki disease can follow by a few days or weeks a wide range of infection involving numerous viruses such as EBV, MERS- and SARS-CoV-1, H1N1 influenza and other respiratory illnesses [10].

## What could Kawasaki disease bring to the understanding of COVID-19 post-infective acute myocarditis?

Analogy of the COVID-19 post-infective acute myocarditis with the Kawasaki Disease Shock Syndrome does not only result from a pediatric cognitive bias, but also from a well-described pathophysiology of systemic arteritis seen in the Kawasaki disease. Neutrophils, especially CD14+ CD16+ cells have been identified in arterial wall early in the disease followed by dendritic cells, CD163+ monocytes/macrophages, cytotoxic CD8+ T cells and CD3+ T cells infiltration and subsequent massive production of chemokines and cytokines, especially IL-1 and IL-6. Similarly to the Kawasaki disease, COVID-19 is recognized as a systemic vasculitis affecting not only the lung but all organs, such as the myocardium [11, 12]. How the COVID-19 histopathognomic signature, systemic microangiopathy and thrombosis can be connected to the overt cytokine release and immune cells tissue infiltration seen in the Kawasaki disease? Again, neutrophils infiltration offers a convincing connection. Recent insight on the role of neutrophils capacity to form extracellular traps (NETs) to ensnare pathogens and limit extension of infection was evidenced in severe COVID-19, similarly to what was reported in acute Kawasaki disease [13].

An important trigger of the Kawasaki disease is the development of immune complexes in the circulation. These immune complexes interact with their cellular receptors—the Fcγ receptors—expressed on macrophages, dendritic cells, neutrophils and platelets promoting phagocytosis, degranulation and respiratory burst. In addition, immune complexes can precipitate in tissue leading to local inflammation, complement activation, and organ dysfunction. In contrast to the Kawasaki disease, the role of post-infective immune complexes has not yet been demonstrated in COVID-19 patients [10], but published data strongly suggest a COVID-19 post-infective component [1, 2]. In the published pediatric series [1–6], patients with acute myocarditis had either a SARS-CoV-2 detection or positive serology with IgG present in the great majority, while no other causes of myocarditis were identified. The expected SARS-CoV-2 antibody response is first an increase in immunoglobulin M, immediately followed by immunoglobulin G with a peak between 17 and 22 days after symptoms onset. By analogy to Kawasaki disease, we cannot exclude that formation of immune complexes play a role in the development of COVID-19 myocarditis. In the published series [1–6], most children with COVID-19 post-infective acute myocarditis were successfully treated with intravenous immunoglobulins and aspirin, similarly to what is recommended for the Kawasaki disease [1–6]. Immunomodulatory effects of immunoglobulins are well described and known to dampen hyperinflammation, and attenuate the immune complex-mediated response seen in patients with Kawasaki disease [10]. Anti-inflammatory dose of aspirin is used in the first 15 days, and then continued at an anti-aggregative regimen for 2 months if coronary arteries failed to show aneurysm. No patients had coronarography as in Kawasaki disease aneurysm are rare and usually occurs in the first coronary segment which is easily assessed by echocardiography or coronary scan. Interestingly, few patients with COVID-19 post-infective myocarditis with incomplete response immunoglobulins received subsequently IL-1 receptor antagonist (Anakinra) therapy and were eventually cured [1, 2]. Targeting IL-1 pathway, especially with massive inflammation, could emerge as a potential therapy in severe COVID-19 patients [14].

## Is COVID-19 post-infective acute myocarditis limited to children and adolescents?

Although many clinical similarities exist with Kawasaki disease, COVID-19 post-infective myocarditis should be considered as a potential complication of COVID-19 that may not be limited to children. We cannot exclude that during the SARS-CoV-2 pandemic, some adult patients with isolated or predominant acute heart failure and negative SARS-CoV-2 PCR would have experienced a post-infective complication of COVID-19. Systematic serology in such a context could help in diagnosing this complication.

## How should we further proceed with COVID-19 post-infective acute myocarditis research?

Current knowledge on Kawasaki disease and the numerous similarities between both clinical entities may help us in establishing research hypothesis (Table 1). Probability of facing the emergence of COVID-19 post-infective complications is high, and the occurrence of post-infective acute myocarditis outbreak urges us to set a systematic clinical, biological and echocardiographic follow-up of all patients who developed COVID-19.

**Table 1:** What is known and how to further proceed with COVID-19 post-infective myocarditis?

	What is known?	What need to be clarified?
Diagnosis		
Post-infective COVID-19 acute myocarditis diagnosis	Positive SARS-CoV-2 serology [1, 2] SARS-CoV-2 virus detection in nasopharynx may be negative [1, 2] Elevated D-Dimer, ferritin, fibrinogen, and CRP (IL-6), troponins, NT-proBNP [1, 2] Lung CT scan may be suggestive of COVID-19 [1, 2] Abdominal symptoms [1, 2] Clinical signs and symptoms compatible with Kawasaki disease [1, 2]	SARS-CoV-2 virus detection in feces may be prolonged Systemic organ involvement: heart, kidney, liver, polyserositis
Investigations		
Circulating cell phenotyping	No available data on post-infective COVID-19	Multiple phenotypic signatures have been suggested in COVID-19, but currently there is a significant requirement of data and correlation with prognosis
Circulating immunoglobulins (quantitative and subclasses)	No available data on post-infective COVID-19	Normal Ig level is seen in COVID-19, although some report suggest elevated circulating immunoglobulins
Cardiac imaging	Sub-epicardial edema (T1 gadolinium, T2-weighted) is seen on cardiac magnetic resonance	Defining cardiac magnetic resonance semiology (2018 Lake Louise Criteria) and kinetics
Autopsy—myocardial biopsy	Presence of lymphocytic myocarditis^a^ Vascularitis and microangiopathy may be present^a^	Coronary microangiopathy Immuno-histochemical analysis of coronary arteries, incl. cells phenotypes (neutrophils, cytotoxic CD8 + Lymphocytes, dendritic cells, macrophages)
Therapy		
Intravenous immunoglobulins	Similarly to Kawasaki disease, COVID-19 post-infective acute myocarditis respond to IVIg in most cases unless heart failure may not support important volume transfusion [1, 2]	Controlled trials comparing hemodynamic tolerance of IVIg and IL-1 receptor antagonist is warranted
IL-1 receptor antagonist	Limited experience suggests that use of Anakinra is well tolerated and clinical response rapid. It may be an alternative to IVIg in depressed myocardial function or in addition if symptoms are refractory to IVIg [1, 2]	

SARS-CoV-2, severe acute respiratory syndrome coronavirus 2; CRP, C-reactive protein; BNP, brain natriuretic peptide; cluster of differentiation-8; IL-6, interleukin 6; COVID-19, coronavirus infection disease 2019; Ig, immunoglobulins; CD8, cluster of differentiation 8; IVIg, intravenous immunoglobulins

^a^Autopsy and endomyocardial biopsy findings are issued from COVID-19 patients [7, 8]

## Data Availability

Not applicable

## Abbreviations

SARS-CoV-2: Severe acute respiratory syndrome coronavirus 2
COVID-19: Coronavirus infection disease 2019
MIS-C: Multisystem Inflammatory Syndrome in Children
CD: Cluster of differentiation,
IL: Interleukin
Ig: Immunoglobulins
IVIg: Intravenous immunoglobulins
